# Mature Teratoma Arising in the Fallopian Tube

**DOI:** 10.7759/cureus.86008

**Published:** 2025-06-14

**Authors:** Eri Obata, Kaei Nasu, Kazuaki Shima, Taisuke Morita, Harunobu Matsumoto

**Affiliations:** 1 Department of Obstetrics and Gynecology, Nakatsu Municipal Hospital, Nakatsu, JPN

**Keywords:** fallopian tube, fertility, laparoscopic surgery, mature teratoma, salpingectomy

## Abstract

Mature teratoma of the fallopian tube is extremely rare. Most cases are incidentally diagnosed as originating from the fallopian tube following cesarean section, diagnostic laparoscopy, or examination of a surgical specimen. Here, we present a case of mature teratoma of the fallopian tube identified during laparoscopic surgery. A 5-cm mature teratoma was detected in the pelvic cavity of a 30-year-old Filipino female using transvaginal ultrasonography, computed tomography, and magnetic resonance imaging (MRI). Laparoscopy revealed a cystic tumor arising from the isthmus of the right fallopian tube, macroscopically distinct from the normal right ovary. The patient underwent laparoscopic right salpingectomy. Histologic analysis confirmed a benign, mature teratoma of the fallopian tube. Gynecologic surgeons should be aware of this rare entity, as it may be encountered incidentally during surgery and influence intraoperative decision-making.

## Introduction

Mature teratomas originate from germ and pluripotent embryonic stem cells and comprise well-differentiated tissues derived from at least two of the three germ layers: endoderm, mesoderm, and ectoderm [1]. These tumors are typically unilocular but may be multilocular, containing tissues such as hair, skin, teeth, sebaceous material, cartilage, bone, salivary glands, and nerve tissue in varying proportions [1]. They are the most common benign ovarian tumors in females of reproductive age, peaking between 20 and 40 years and accounting for 16-20% of all ovarian tumors [1].

By contrast, mature teratomas of the fallopian tube are exceedingly rare, with fewer than 100 cases reported in the English-language literature [2-5]. The incidence and histogenesis remain unclear due to their rarity [1,6].

These tumors are often misdiagnosed as ovarian teratomas on imaging modalities such as ultrasonography, computed tomography, or magnetic resonance imaging (MRI) prior to surgery [6-9]. Definitive diagnosis frequently occurs incidentally during cesarean section, diagnostic laparoscopy, or histologic examination [2,4,6].

Herein, we report a rare case of mature teratoma of the fallopian tube diagnosed intraoperatively during laparoscopic surgery.

## Case presentation

**Figure 1:**
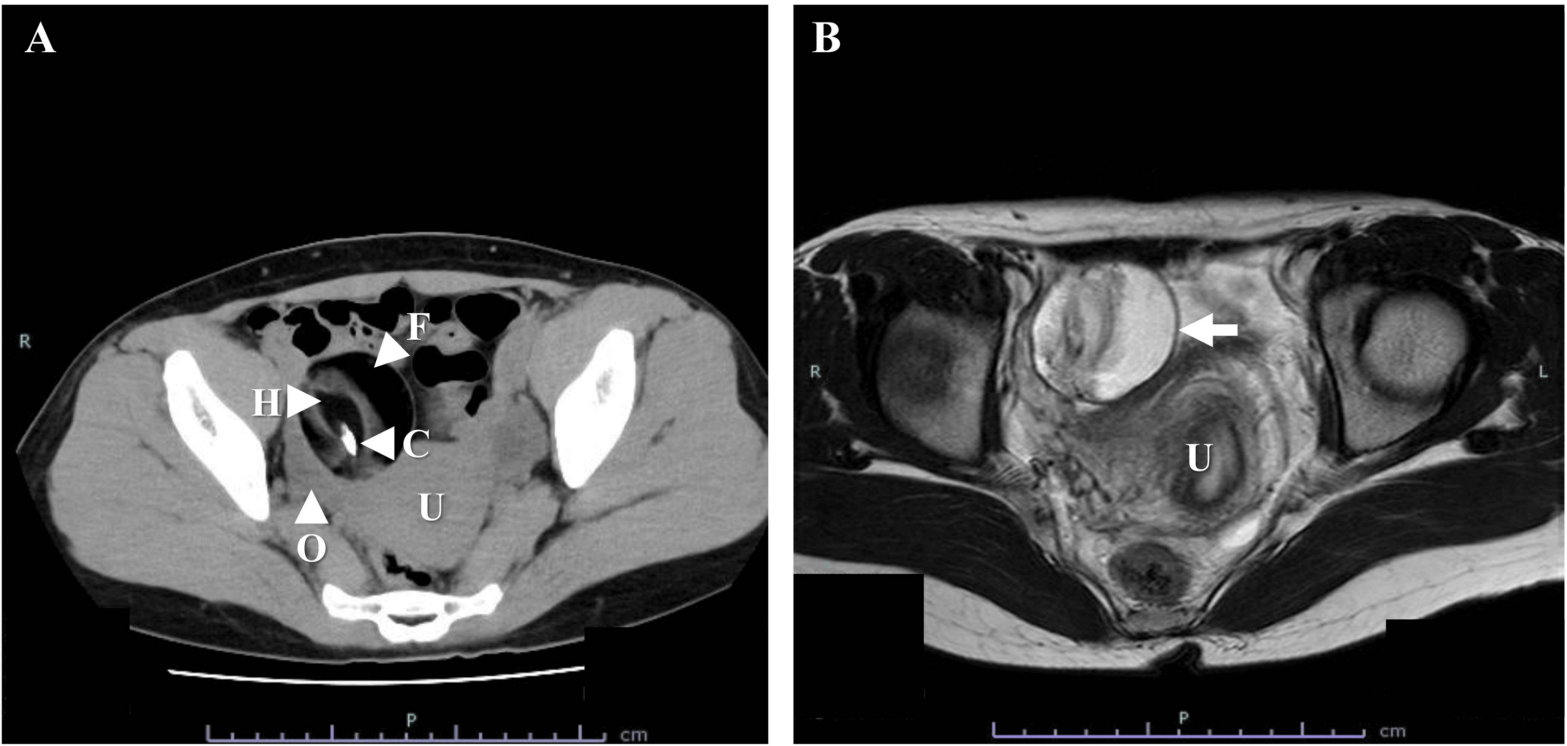
CT and MRI findings of the pelvic tumor (A) CT revealed a 5-cm, well-circumscribed, unilocular cystic lesion in the right adnexal region. The lesion contained fatty fluid, calcification, and a hairball. (B) MRI (T2-weighted images) showed a 5-cm, well-circumscribed, high-intensity mass in the right adnexal region (arrow). Its continuity with the right ovary was unclear. No ascites was observed. C: calcification; F: fatty fluid; H: hairball; O: right ovary; U: uterus

A 30-year-old nonpregnant Filipino female (gravida 1, para 1) saw her family doctor with the complaint of dizziness and transient loss of consciousness, and a pelvic mass was pointed out by computed tomography. Then, the patient was referred for evaluation of a pelvic mass. A 5-cm mature teratoma was identified in the pelvic cavity via transvaginal ultrasonography, computed tomography, and MRI (Figure 1).

Continuity with the right ovary was unclear. Serum levels of cancer antigen (CA)19-9 and CA125 were 40.7 and 18.0 U/mL, respectively.

**Figure 2:**
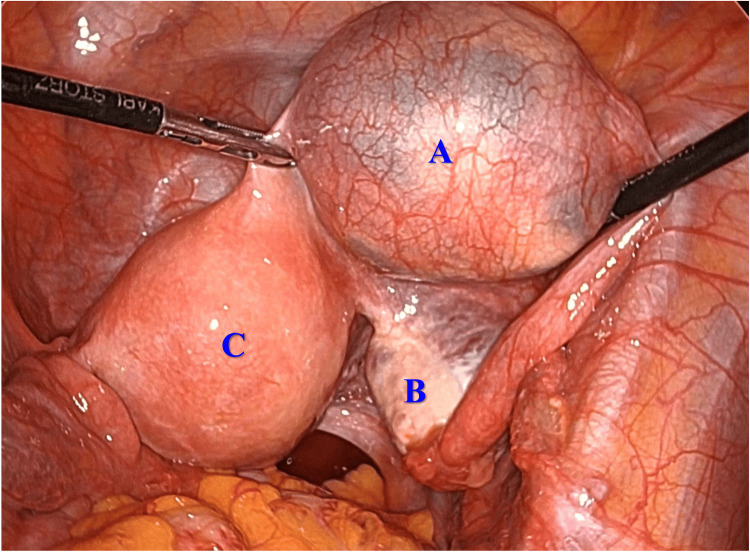
Laparoscopic findings Laparoscopy demonstrated a cystic tumor arising from the isthmus of the right fallopian tube, macroscopically separated from the right ovary. (A) Cystic tumor of the right fallopian tube; (B) right ovary; (C) uterus.

The patient underwent laparoscopic surgery. A cystic tumor was found in the isthmus of the right fallopian tube and was macroscopically separate from the right ovary (Figure 2).

**Figure 3:**
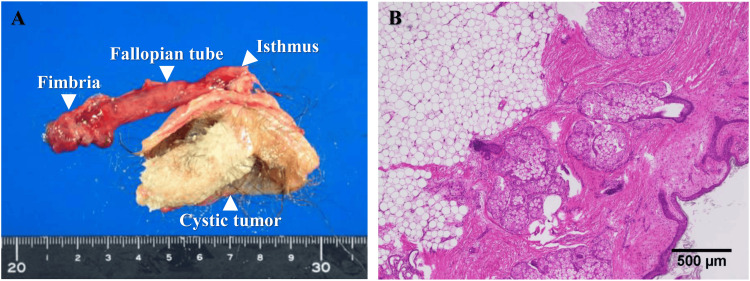
Macroscopic and microscopic findings of the resected tumor (A) The resected tumor from the right fallopian tube consisted of a single cyst containing adipose tissue and a hairball. (B) Hematoxylin and eosin staining showed the cyst was lined by squamous epithelium with skin appendages. The tumor was distinct from the luminal structure of the fallopian tube. No immature elements were detected.

Both ovaries were normal. Laparoscopic right salpingectomy was performed using an EndoCatch (US Surgical, Norwalk, CT), and the tumor was removed intact from the peritoneal cavity. Histologic evaluation confirmed a well-circumscribed, benign mature teratoma (Figures 3A, 3B).

The postoperative course was uneventful. At one-year follow-up, the patient remained asymptomatic without recurrence. Written informed consent was obtained for publication of this case.

## Discussion

Mature teratomas of the fallopian tube are typically asymptomatic, but they may cause subfertility, infertility, irregular menstruation, menorrhagia, abnormal vaginal discharge, postmenopausal bleeding, ectopic tubal pregnancy, adnexal masses, or lower abdominal discomfort [2,3,6-8,10]. A tumor within the fallopian tube may compromise fertility by obstructing fertilization. Rupture with subsequent acute peritonitis has also been reported [11]. In our present case, there were no symptoms related to mature teratoma of the fallopian tube. The tumor was found by chance using computed tomography.

These tumors are generally solitary, unilocular, and most frequently located in the ampullary portion of the fallopian tube [2,3,7,10]. Reported cases involve females aged 17-67 years, with most being nulliparous [2,3]. Mature teratomas are slightly more prevalent in the right fallopian tube than in the left [3,10]. Teratoma can develop in any region of the fallopian tube, but the ampulla and isthmus are the most common sites [3,10]. Intraluminal teratomas predominate but may also be located in intramural or serosal layers [3,10]. In our present case, the tumor was located in the subserosal layer of the isthmus.

Because the anatomical distance between the fallopian tube and the ovary is very close, mature teratomas of the fallopian tube are often misdiagnosed as ovarian teratomas on imaging modalities such as ultrasonography, computed tomography, or MRI prior to surgery [6-9]. Preoperative diagnosis of mature teratomas of the fallopian tube is very difficult [2,4,6].

Surgical excision via laparoscopy or laparotomy is the standard treatment [3]. Prognosis is favorable with complete resection. Salpingectomy is recommended for patients who do not wish to have a baby. Conservative surgery, such as cystectomy, may be considered in nulliparous females when the tumor is located in the fimbrial end. To date, no cases of malignant transformation of mature teratoma of the fallopian tube have been reported [3]. Tumor markers such as CA125 and CA19-9 are usually within normal limits in these cases, though elevations have been documented, as in the present case [12].

## Conclusions

This case highlights a rare occurrence of mature teratoma of the fallopian tube diagnosed during laparoscopic surgery. Gynecologic surgeons should consider this possibility during intraoperative assessment of pelvic masses.
